# Exploring traditional aus-type rice for metabolites conferring drought tolerance

**DOI:** 10.1186/s12284-017-0189-7

**Published:** 2018-01-25

**Authors:** Alberto Casartelli, David Riewe, Hans Michael Hubberten, Thomas Altmann, Rainer Hoefgen, Sigrid Heuer

**Affiliations:** 1 School of Agriculture, Food and Wine, Waite Campus, The University of Adelaide, Adelaide, SA Australia; 2 Julius Kühn-Institute (JKI), Federal Research Centre for Cultivated Plants, Institute for Ecological Chemistry, Plant Analysis and Stored Product Protection, Berlin, Germany; 3 Max Planck Institute of Molecular Plant Physiology, Potsdam-Golm, Germany; 4 Rothamsted Research, Harpenden, UK

**Keywords:** Rice, Aus-type landraces, Metabolites, Allantoin, Drought tolerance, Roots, Spikelets, Genetic diversity

## Abstract

**Background:**

Traditional varieties and landraces belonging to the aus-type group of rice (*Oryza sativa* L.) are known to be highly tolerant to environmental stresses, such as drought and heat, and are therefore recognized as a valuable genetic resource for crop improvement. Using two aus-type (Dular, N22) and two drought intolerant irrigated varieties (IR64, IR74) an untargeted metabolomics analysis was conducted to identify drought-responsive metabolites associated with tolerance.

**Results:**

The superior drought tolerance of Dular and N22 compared with the irrigated varieties was confirmed by phenotyping plants grown to maturity after imposing severe drought stress in a dry-down treatment. Dular and N22 did not show a significant reduction in grain yield compared to well-watered control plants, whereas the intolerant varieties showed a significant reduction in both, total spikelet number and grain yield. The metabolomics analysis was conducted with shoot and root samples of plants at the tillering stage at the end of the dry-down treatment. The data revealed an overall higher accumulation of N-rich metabolites (amino acids and nucleotide-related metabolites allantoin and uridine) in shoots of the tolerant varieties. In roots, the aus-type varieties were characterised by a higher reduction of metabolites representative of glycolysis and the TCA cycle, such as malate, glyceric acid and glyceric acid-3-phosphate. On the other hand, the oligosaccharide raffinose showed a higher fold increase in both, shoots and roots of the sensitive genotypes. The data further showed that, for certain drought-responsive metabolites, differences between the contrasting rice varieties were already evident under well-watered control conditions.

**Conclusions:**

The drought tolerance-related metabolites identified in the aus-type varieties provide a valuable set of protective compounds and an entry point for assessing genetic diversity in the underlying pathways for developing drought tolerant rice and other crops.

**Electronic supplementary material:**

The online version of this article (10.1186/s12284-017-0189-7) contains supplementary material, which is available to authorized users.

## Background

To meet the increasing demand for food due to an increasing world population, future agricultural systems need to become more productive and, at the same time, more resource-use efficient and sustainable. Rice (*Oryza sativa* L.) is currently the main source of calories for more than half of the world’s population and considerable breeding efforts are undertaken globally to increase the yield and yield potential of rice (for a recent review see Khan et al. 2015). However, there is also considerable scope for increasing yield by closing yield gaps, i.e., reducing the difference between the actual yield and the attainable yield, which is generally determined by light intensity and temperature, as well as nutrient- and water-availability. Modelling of yield gaps caused by water and nutrient limitations showed that closing the yield gap in maize, wheat and rice to 75% of the attainable yield would equal a 29% increase in global production (Mueller et al. 2012).

For closing such yield gaps it will be important to improve water and farm management, but equally important to develop crops that maintain high yield under adverse conditions, such as heat and drought or submergence, and increasing pest and disease pressure. The enhancement of drought tolerance in rice is one of the key challenges due to more frequent and more severe drought events caused by climate change (Porter et al. 2014; Lesk et al. 2016) and the need to reduce water consumption of rice production. First drought-tolerant rice varieties developed by marker-assisted selection (MAS) are now being released conferring yield advantages under drought across different environments of about 11% on average compared with the control (Swamy et al. 2013). Likewise, submergence tolerant Sub1-rice varieties had a yield advantage of more than 50% in submergence-prone regions across India (Mackill et al. 2012). This shows the potential impact of breeding for stress tolerance.

In recent years, a specific group of rice, so called aus-type rice, has been discovered as a valuable source of stress tolerance. Aus-type rice is closest related to indica-type rice but constitutes a distinct genetic group (McNally et al. 2009). These landraces have evolved and are still cultivated under environmental stress conditions in India and Bangladesh (Londo et al. 2006) and therefore have developed and preserved tolerance mechanisms for a diversity of stresses. For example, the submergence tolerance gene *OsSUB1A* mentioned above and the phosphorus (P) -starvation tolerance gene *OsPSTOL1* have both been identified from aus-type rice varieties (Xu et al. 2006; Gamuyao et al. 2012) and the aus-type variety N22 has been described as one of the most heat-tolerant rice cultivars currently known (Li et al. 2015; González-Schain et al. 2016). The variety Dular showed the highest P uptake under low-P field conditions (Wissuwa and Ae 2001) and consistently ranked highest in a drought study showing the least yield reduction over multiple seasons compared to other genotypes (Henry et al. 2011).

Aus-type rice is therefore highly valuable for breeding applications as a source of novel tolerance traits but also for gene discovery research. With the availability of an N22 *de-novo* reference genome (https://pag.confex.com/pag/xxiv/webprogram/Paper21395.html) and “omics” technologies it is now possible to assess aus-type rice at the molecular level and more easily gain access to genes and pathways that are specific to this group of rice. This will be important since so far molecular studies on stress tolerance have been predominantly carried out using the japonica type variety Nipponbare, for which a reference genome and genetic resources are available. However, Nipponbare is a modern irrigated variety and as such intolerant to drought and other abiotic stresses. Genes and pathways that are stress responsive in Nipponbare are therefore representative of an intolerant response and might be distinct from those in tolerant genotypes. In fact, important genes such as *OsSUB1A* and *OsPSTOL1*, or *Deep Root 1* (DRO1) and the S*NORKEL* deep water rice genes are not present in Nipponbare (Xu et al. 2006; Hattori et al. 2009; Gamuyao et al. 2012; Uga et al. 2013) and a comparative genome analysis of the aus-type variety DJ123 with Nipponbare and an indica genome (IR64) identified more than 600 genes that were specific to the aus-type variety (Schatz et al. 2014).

Metabolomics is regarded as the most transversal among the “*omics”* technologies mainly because it is not dependent on the availability of reference genomes and because it is untargeted and as such comprehensive, high throughput and facilitates the discovery of novel biomarkers (Beckles and Roessner 2012). Metabolites provide a direct readout of the physiological status of plants, reflecting the end products of the effect of environmental factors and the genetically determined, physiological and developmental responses of plants regulated by highly complex signalling and posttranslational processes. Therefore, metabolomics is closer to the phenotype than transcriptomics or proteomics alone (Beckles and Roessner 2012). Mapping of metabolites has already been applied to identify quantitative trait loci (QTL e.g. Matsuda et al. 2012; Hill et al. 2015) and potentially metabolites associated with a given trait of interest can be used as a screening and phenotyping tool in breeding programs, such as for quality traits in rice (Redestig et al. 2011; Matsuda et al. 2012). A comparative study of metabolomics and whole-genome SNP markers in maize has furthermore shown that metabolite profiles can predict the heterotic potential and yield of adult hybrid plants (Riedelsheimer et al. 2012).

In this study, we have conducted an untargeted, factorial metabolome analysis to compare the drought response of two tolerant aus-type varieties (Dular and N22) with two modern irrigated rice varieties (IR74 and IR64) grown under well-watered and dry-down conditions in soil. The main objective was to identify metabolites and their underlying pathways that are associated with drought tolerance, i.e., metabolites that show a distinct drought response in the aus-type rice varieties.

## Methods

### Plant material and growth conditions

Seeds of the two aus-type varieties (N22: IRGC19379; Dular: IRGC32561) and two indica-type irrigated varieties (IR64: IRGC66970; IR74: IRGC76331) used in this study were derived from IRRI’s International Rice Genebank Collection (IRGC) in the Philippines.

Plants were grown in a glasshouse at IRRI (Los Banos, Laguna, Philippines) under the natural tropical conditions from September to December. Pots were filled with 6 kg of sifted local soil (anthraquic Gleysols) with basal fertilizer application equivalent to 45-30-20 kg ha^−1^ N-P-K. In total, 48 plants were grown for each genotype with two plants in each pot.

All pots were kept well-watered (WW) until 18 days after sowing (DAS), when water was withheld from half of the pots for the dry-down drought (D) treatment. Leaf rolling in D stressed plants occurred at 32 DAS in all genotypes and roots and shoots of 16 plants (8 pots) for each genotype were harvested. Roots and shoots of the same number of plants were harvested from WW controls at 33 DAS. For each genotype and treatment, 4 pots were harvested during the morning and four in the afternoon to account for time-of-day variation in metabolite compositions. Because the D-treated soil was very hard, pots had to be soaked in water for about 30 min before plants could be removed from the soil without damaging the root system. Roots, still attached to the shoot, were then washed with tap water on a sieve and rinsed twice with de-ionized water. Root and shoot length was measured with a ruler before shoots and roots were separated and frozen in liquid nitrogen. Samples were stored at -80 °C until they were further processed for metabolite analysis.

The remaining plants (8 plants in 4 pots for each genotype and treatment) were grown to maturity to assess phenotypic differences in the effect of the D treatment among the selected rice genotypes. D stressed plants were re-watered at 32 DAS for 2 days and water was withheld for a second dry-down until leaf rolling, which occurred at 42 DAS. From then on, the soil was kept flooded until plant maturity with a second fertilizer application (same as above) at 46 DAS. Pots with the WW control plants were kept flooded at all times.

In summary, the sample sets for the metabolite analysis and the phenotyping at maturity consisted of 15–16 biological replicates for each the two treatments (WW and D) and tissues (shoot and root) for each of the four genotypes (N22, Dular, IR64 and IR74).

### GC-MS and IC analysis and data acquisition

Metabolites were extracted from 50 ± 5 mg fresh weight, measured and processed as described in Riewe et al. (2012 and 2016) using a LECO Pegasus HT mass spectrometer (LECO, St. Joseph, MI, USA) hyphenated with an Agilent 7890 gas chromatograph (Agilent, Santa Clara, CA, USA) and a Gerstel MPS2-XL autosampler (Gerstel, Muelheim/Ruhr, Germany). Eighty-nine known and 226 unknown metabolites were quantified in split-less mode. Lactate, malate, fructose, glucose and sucrose were quantified using split injections (1:50). Data were normalized regarding sample weight, measurement day and median of the respective metabolite per analysed batch. Outliers (more or less than replicate median +/−2xSD) were removed. For the WW shoots samples, a cluster of 28 samples was also excluded from the analysis due to technical problems during the sample preparation and the number of replicates for these samples was therefore reduced to 8–11. The full list of annotated metabolite peaks is provided as Additional file 1: Table S1.

### Ion chromatography analysis

A subfraction of the polar phase containing polar metabolites and inorganic ions was filtered using an Ultrafree MC 5000 MC NMWL filter unit (Millipore). Subsequently, anions and cations were analyzed by high-performance anion- and cation-exchange chromatography with conductivity detection facilitated by a Dionex ICS-3000 system as described in detail in Schmidt et al. (2013).

### Data analysis

For the identification of tolerant-specific metabolites, the normalised data was log10 transformed to improve normality and analysed by a two factorial ANOVA with interaction, where the factors were treatment (WW or D) and genotype. For this purpose, the tolerant aus-type varieties Dular and N22 were combined into a tolerant group and IR64 and IR74 into a sensitive group. A Bonferroni correction (Broadhurst and Kell 2006) was applied to account for multiple testing.

For the pathway map, Student’s *t*-test analysis coupled with Bonferroni correction was performed between treatment and control values to highlight individual metabolite trends and metabolite networks were constructed using KEGG pathway maps web tool (http://www.genome.jp/kegg/).

## Results

### Phenotypic effect of drought stress in tolerant and intolerant rice genotypes

For this study we have chosen two representative aus-type varieties (Dular and N22) and two irrigated indica-type rice varieties (IR64 and IR74) to assess differential responses to water deficit at the metabolite level. Plants were grown in soil-filled pots under well-watered (WW) and dry-down drought (D) stress conditions until leaf rolling. A parallel set of WW plants and plants re-watered after drought stress was grown to maturity for yield component analysis.

**Fig. 1:**
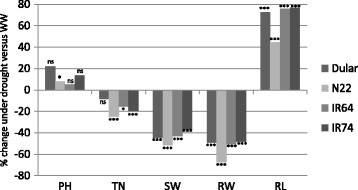
Phenotypic data at the vegetative stage of four rice genotypes used for metabolite profiling. Four rice genotypes representing stress-tolerant aus-type varieties (Dular, N22) and intolerant indica-type irrigated rice varieties (IR64, IR74) were grown in soil-filled pots under well-watered and dry-down conditions. Root and shoot samples for metabolite analysis were harvest at 33 days after seeding. PH = plant height; TN = tiller number; SW = shoot fresh weight; RW = root fresh weight; RL = length of longest root. Asterisks indicate significant differences between WW and drought conditions: * = *p* < 0.05; ** = *p* < 0.01; *** = *p* < 0.005; ns = not significant

All genotypes responded to the applied drought conditions by significantly increasing length of the longest root by 40% to almost 80% (Fig. 1). At the same time, root fresh weight (FW) was decreased by about 50%. Of the four genotypes, N22 showed the least root elongation and the highest reduction in root FW (Fig. 1). No differences in root length (longest root) were observed at the time of sampling for the metabolite analysis and the aus-type varieties had only slightly longer roots on average (Dular: 26.3 cm; N22: 27.5 cm) compared with the irrigated varieties (IR64: 25.7 cm; IR74: 23.6 cm) (data not shown).

Plant height was generally not affected by the treatment in any genotype but a significant reduction in tiller number by about 20% was observed in N22, IR64 and IR74 (Fig. 1), which corresponded to a reduction by about 2 tillers (data not shown). All genotypes accordingly showed a significant reduction in shoot FW by about 40% (Fig. 1).

In plants grown to maturity, likewise no effect of the treatment on plant height was observed and tiller number of re-watered plants remained about 20% lower compared with WW controls in all genotypes (Additional file 2: Figure S1). In contrast, shoot DW recovered in all genotypes, except in IR64 which showed 20% lower shoot DW compared to the WW control. Likewise, reduction in root DW in re-watered plants remained highest in IR64 (> 40%) compared to about 20% reduced root DW in the other genotypes (Additional file 2: Figure S1).

Recording of the flowering time showed that the D treatment delayed flowering by four (IR64) to 7 days (Dular) and up to 10 days (N22, IR74). However, spikelet fertility and grain yield clearly differentiated between tolerant and intolerant genotypes. Whereas the aus-type varieties Dular and N22 did not show a significant reduction in the number of filled spikelets (grain) compared to the WW controls, the D treatment significantly reduced the total number of spikelets and the number of grains per panicle in IR64 and IR74 (Additional file 2: Figure S2). Interestingly, total spikelet number per panicle increased significantly in Dular, which compensated for the low spikelet fertility (62%) in the re-watered plants. Spikelet fertility in the other genotypes was largely unaffected by the D treatment, i.e., the reduced yield in IR64 and IR74 is due to a reduced spikelet number not due to reduced fertility (Additional file 2: Figure S2). This long-term negative effect of vegetative drought on yield appeared to be independent of phenology since IR74 flowered much later (91 DAS) than IR64 (63 DAS), which was closer to the aus-type varieties (Dular: 68 DAS; N22: 58 DAS).

### Genotype and treatment effect on metabolite profiles

**Fig. 2:**
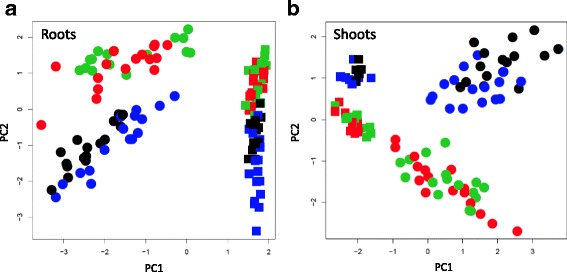
PCA analysis of metabolites in roots and shoots. The first two principal components of root (**a**) and shoot (**b**) samples of four rice genotypes and two treatments (well-watered and dry down) are shown. Squares = samples from well-watered plants; circles = samples from plants exposed to a dry-down treatment; black = Dular; blue = N22; red = IR64; green = IR74

Root and shoot material of plants harvested from WW and D plants was analysed by ion chromatography-conductivity and gas chromatography-mass spectrometry (GC-MS). The combined dataset comprised a total of 328 metabolites and inorganic ions, of which 102 could be annotated. The metabolite data sets derived from root and shoot samples of the four genotypes were subjected to principal component analysis (PCA) to score for general trends (Roessner et al. 2001). This analysis showed that the first two principal components are sufficient for a clear separation of the genotypes according to treatment (PC1) and drought response (PC2) (Fig. 2a, b; Additional file 1: Table S1 for PC loadings). For both, roots and shoots, PC1 separates metabolome data sets of WW from drought stressed plants and in both cases samples derived from WW plants were less variable than those from the dry-down plants, suggesting less homogenous growth conditions as can be expected during dry-down of large pots. For the roots, PC2 separated the tolerant varieties Dular and N22 from the drought-intolerant indica varieties (IR64 and IR74) at comparable levels in both, the WW and the dry-down samples (Fig. 2a). PC2 also separated the aus-type and the indica varieties in the shoot samples (Fig. 2b), however, due to specific changes in the sensitive cultivars IR64 and IR74, separation of the drought samples in shoots is higher. Hierarchical clustering of all metabolites confirmed the PC analysis and clearly separated between genotypes and treatment in both, shoots and roots (Additional file 2: Figures S3 and S4). Other parameters, such as the spatial distribution of pots in the greenhouse, time and day of harvest and measurements was tested but did not show an effect (data not shown). The PC analysis therefore provided sufficient evidence that the metabolome data are indicative of the genetic differences between the genotypes and of the treatment effect and hence suitable for scoring specific diagnostic metabolite signatures.

### Metabolic response of the primary metabolism of shoot and root subjected to drought

**Fig. 3:**
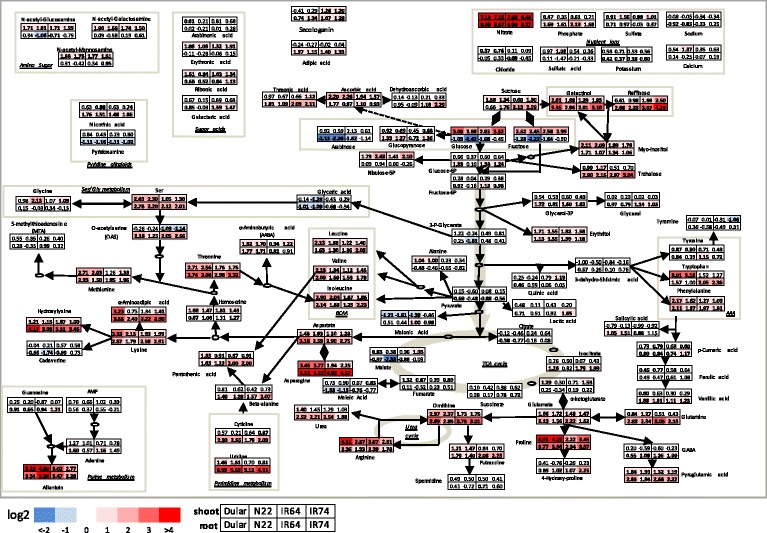
Metabolic changes in the primary metabolism in shoots and roots in response to drought. Variation in metabolite levels in shoots and roots are presented as a log_2_ ratio per variety, pairwise by stress versus control conditions. Bold values indicate significant differences between drought and well-watered conditions (Student t-test with Bonferroni correction, *P* < 0,05). Colour coding indicates significantly different metabolites with a log2FC higher or lower than one (two-fold in linear scale)

Metabolic and ion profiling allowed the identification and annotation of 102 molecules including amino acids, sugars, organic acids and nutrient ions. The results are summarised in the pathway map shown in Fig. 3. The relative abundance of each metabolite in the individual genotypes and tissues is colour coded and presented as the log_2_ value of the fold change (log2FC) between stress and control conditions (see M & M for details). Overall, a large number (83) of metabolites showed a response to the drought treatment by either increasing (positive log2FC) or reducing (negative log2FC) the levels under drought (Fig. 3).

**Fig. 4:**
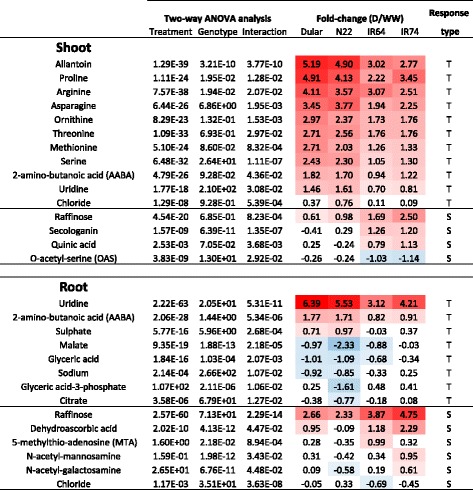
Drought-responsive metabolites with differential accumulation in tolerant and sensitive rice genotypes. Metabolites with significant differences in shoots and roots between the analysed rice genotypes based on a Two-Way ANOVA are listed. Red and blue color shading highlight the degree of postive and negative-fold change in response to the drought treatment. Metabolites with higher fold-change in the tolerant or intolerant genotypes, respectively, were classidified as tolerant-specific (T) or sensitive-specific (S)

**Fig. 5:**
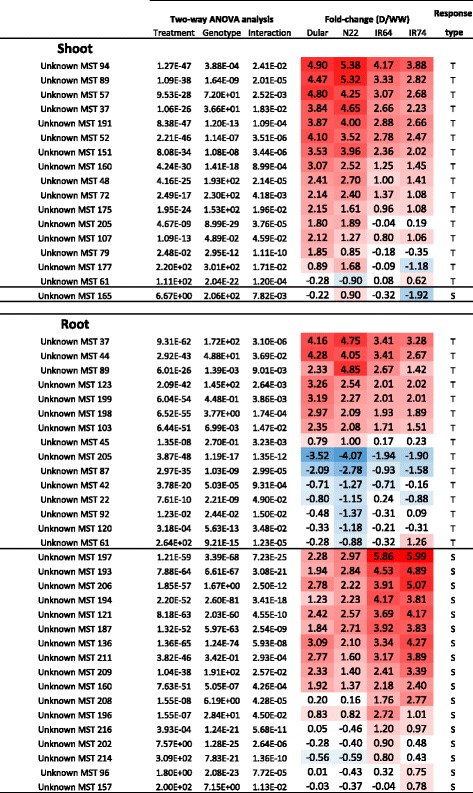
Unknown drought-responsive metabolites in tolerant and sensitive rice genotypes. Metabolites without annotation but significant differences in shoots and roots between the analysed rice genotypes based on a Two-Way ANOVA are listed. Red and blue color shading highlight the degree of postive and negative-fold change in response to the drought treatment. Metabolites with higher fold-change in the tolerant or intolerant genotypes, respectively, were classidified as tolerant-specific (T) or sensitive-specific (S)

To reduce complexity, only those up- and down-regulated metabolites presenting a log2FC larger or smaller than 1 (*P* < 0.05) were considered as significantly altered under the applied experimental conditions. This was further supported by an estimation of the theoretical possible accumulation of metabolites simply caused by the reduction in FW under drought, which was 0.86-log2FC in shoots and 1.14-log2FC in roots. The median accumulation over all the metabolite features identified was 0.74-log2FC in shoots and 0.93-log2FC roots. While this is close to the theoretical enrichment, most of the identified metabolites were well above that threshold as shown in Figs. 4 and 5.

Of all analysed metabolites, nitrate (NO_3_^−^) showed the highest accumulation in the shoots (7.2-log2FC) as well as in the roots (9.3-log2FC). In contrast, phosphate (PO_4_^−3^) also increased in roots (up to 2.1-log2FC) but did not show any significant changes in shoots. The remaining nutrient ions (Cl^−^, SO_4_^−2^, H_2_SO_4_, Na^+,^ K^+^, Ca^++^) did not show major significant differences between genotypes or treatment response.

A range of sugars were affected by the treatment with the majority showing a positive log2FC in both, roots and shoots (Fig. 3). Sucrose, which is thought to play a key role in osmotic adjustment (Lemoine et al. 2013), increased only slightly in shoots but up to 2.6-log2FC in roots, whilst glucose and fructose positively accumulated in shoots (up to 3.3-log2FC) but were reduced in roots (up to −2.4-log2FC). Sugars belonging to the raffinose family oligosaccharides (RFO), known to have a protective role under stress (Nishizawa et al. 2008), also increased significantly (raffinose: 4.8-log2FC; galactinol: 3.4-log2FC; myo-inositol: 2.1-log2FC) together with the polyol erythritol (2-log2FC). Interestingly, other sugars were responsive specifically in roots but not in shoots, such as trehalose (3-log2FC) and arabinose (−2.3-log2FC). In contrast, the phospho-sugar ribulose-5-phosphate showed a positive accumulation (2.5-log2FC) specifically in shoots.

Metabolite levels of intermediates of the glycolysis and the tricarboxylic acid cycle (TCA) were not strongly affected by the treatment (Fig. 3). Although in roots, reduction under drought was observed for 3-P-glycerate (−1.6-log2FC, N22-specific), malate (−2.33-log2FC, N22-specific), glyceric acid (−1-log2FC, Dular and N22-specific), whilst accumulation for isocitrate (up to 2-log2FC) was observed in Dular, IR64 and IR74.

Amino acids (AA) were the class of primary metabolites that presented the most widespread response, which is in agreement with other studies conducted on abiotic stresses (e.g. Bowne et al. 2011; Planchet et al. 2011; Witt et al. 2012) and an important role of proline for drought tolerance in rye grass was already suggested 60 years ago (Kemble and Macpherson 1954).

In our study, AA belonging to the glutamate family showed a strong positive accumulation in response to drought in both, shoots and roots, with the highest log2FC observed for proline (4.9-log2FC) followed by arginine (4.1-log2FC), the intermediate ornithine (3.8-log2FC) and glutamate (2.2-log2FC). In contrast to these AA, glutamine showed a positive accumulation (3.1-log2FC) mainly in roots (Fig. 3). Increased levels were also observed for other metabolites related to the family, such as GABA (2-log2FC) and urea (2.6-log2FC). AAs belonging to the aspartate family overall increased under drought in both, shoots and roots, with asparagine increasing the most (5.5-log2FC), followed by threonine (3.7-log2FC), lysine (3.3-log2FC), aspartate (3.2-log2FC) and methionine (2.7-log2FC). Interestingly, aspartate, asparagine and the lysine-related metabolite hydroxylysine showed a relatively higher accumulation in roots than in shoots (Fig. 3). The latter, for example, accumulated up to 4.2-log2FC in roots, while it was only up to 1.7-log2FC in shoots. The branched-chain amino acids (BCAA) presented similar positive accumulations in both tissues under stress (leucine: 2.1-log2FC, valine: 2.2-log2FC, isoleucine: 2.9-log2FC). Within the serine family, serine showed a positive (2.8-log2FC) accumulation in shoots and roots, while the intermediate O-acetyl serine (OAS) increased in roots (2.2-log2FC) but decreased in shoots (−1.4-log2FC), especially in the intolerant genotypes IR64 and IR74 (Fig. 3). Among the aromatic AA, only phenylalanine showed a consistent accumulation (2.2-log2FC) under drought in all genotypes and tissues, while changes in tryptophan were genotype specific with high accumulation in shoots of the tolerant genotypes (>3-log2FC) but in roots of the intolerant genotypes (>2-log2FC). Tissue-specific accumulation under stress was also observed for three amino sugars (N-acetyl-glucosamine, N-acetyl-galactosamine and N-acetyl-mannosamine) which increased specifically in drought stressed shoots (up to 1.9-log2FC). In contrast, the pyridine alkaloids nicotinic acid increased (1.9-log2FC) and pyridoxamine decreased (−1.2-log2FC) specifically in roots.

Metabolites related to nucleotide metabolisms were strongly affected by the drought treatment. Allantoin, indicative of the purine catabolic pathway, increased in roots and shoots about 5-log2FC in the tolerant genotypes and about 3-log2FC in the intolerant genotypes (Fig. 3). Allantoin accumulation was recently reported to increase tolerance under various stress conditions in Arabidopsis (Watanabe et al. 2014; Irani and Todd 2016; Lescano et al. 2016). Pyrimidine-related molecules, such as cytidine and uridine, increased 2.9- and 6.4-log2FC, respectively, but preferentially accumulated in drought-stressed roots.

### Identification of tolerant- and sensitive-specific metabolites

The principal aim of this study was to identify metabolites that are associated with drought tolerance and are therefore specifically responsive in the aus-type varieties N22 and Dular. We have therefore conducted a two-way ANOVA analysis using drought treatment as one factor and tolerance group as the second factor. For the latter, Dular and N22 were combined into a tolerant group and the IR64 and IR74 into a sensitive group (Additional file 3: Table S2) which is also justified by the fact that in the PCA analysis (Fig. 2) no separation within the group of aus-type and indica type varieties could be detected. Overall, in shoots, 79 metabolites were significantly (*P* < 0.05) changed because of the drought treatment, 26 because of the genotype and 15 because of the interaction of the two factors (Additional file 3: Table S2). The latter represent metabolites that accumulated under drought differentially in tolerant and sensitive genotypes and the data are shown in Fig. 4 as log_2_ ratios. Similarly, in roots, 85 metabolites were significantly changed because of the stress, 30 because of the genotype, and 14 as a result of the interaction of the two factors (Fig. 4 and Additional file 3: Table S2). Metabolites were further classified depending on whether the log2FC was higher in the tolerant group or in the drought sensitive group.

A general comparison between the identified metabolites in roots and shoots revealed major differences between the two tissue types. In shoots of the tolerant group, several AA (serine, methionine, asparagine, proline, threonine, arginine and its derivate ornithine) specifically accumulated in response to drought, whereas no AA showed significant interaction between treatment and genotype in roots. Conversely, several organic acids (glyceric acid, glyceric acid 3-phoshate, malic acid and citric acid), which are all components of glycolysis and the TCA cycle, were significant for roots of the tolerant group but not in shoots. In contrast, two metabolites were identified in the tolerant group in both, shoots and roots, namely uridine and 2-amino-butanoic acid (AABA), whereas raffinose was specific to the sensitive groups in shoots and roots (Fig. 4). Chloride was associated with the tolerant genotypes in shoots but with the sensitive genotypes in roots.

**Fig. 6:**
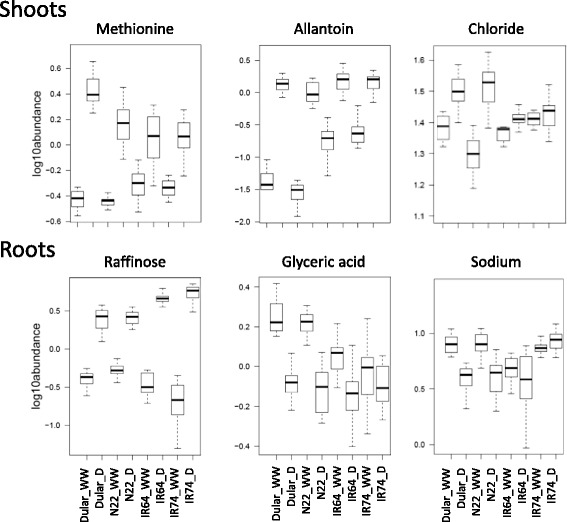
Representative drought responsive metabolites in tolerant and sensitive rice genotypes. Box plots showing the log10 abundance of the metabolites indicated in shoot and root samples of four rice genotypes grown under well-watered (WW) conditions or exposed to a dry-down (D) treatment

Amongst the identified metabolites different drought response pattern were apparent as can be seen in the box plots of representative metabolites from shoots and roots shown in Fig. 6. Generally, three different response types can be distinguished as (i) the magnitude of the response between control and stress (either positive or negative) is higher in the tolerant or sensitive genotypes; (ii) metabolite abundances are different between tolerant and sensitive genotypes under WW conditions rather than under stress; and (iii) the metabolite is responsive to the treatment in only one of the two groups.

Methionine and raffinose are examples of category one, as they showed a higher magnitude of positive change under drought in shoots of the tolerant genotypes and roots of the sensitive genotypes, respectively (Fig. 6). Allantoin is a representative of category two since it accumulated to about the same level in drought-stressed shoots in all genotypes but was less abundant in the tolerant genotypes under well-watered conditions, thus, explaining the higher log2FC in Dular and N22 compared with the sensitive genotypes. The opposite is true for glyceric acid, which was more abundant under WW conditions in roots of the tolerant genotypes compared with IR64 and IR74 but showed no differences under drought. Chloride and sodium are representative of category three since they were not responsive to stress in intolerant genotypes but increased and decreased in N22 and Dular shoots and roots, respectively.

**Fig. 7:**
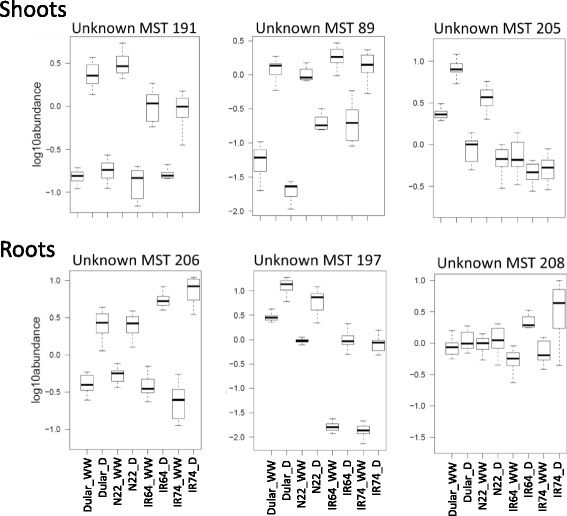
Representative unknown metabolites associated with drought tolerance. Box plots showing the log_10_ abundance of the unknown metabolites indicated in shoot and root samples of four rice genotypes grown under well-watered (WW) conditions or exposed to a dry-down (D) treatment

Although metabolomics is becoming an increasingly popular technique for studying plant stress responses, one of its main current limitations is the large amount of unknown metabolites that are identified during the analysis. For example, in our experiment 226 out of 328 of the registered metabolic features were annotated as unknown. However, despite being unable to place these metabolites in a pathway map, unknowns are still indicative of the genetic diversity between genotypes and their contrasting stress responses. Applying the same analysis as conducted for the annotated metabolites, we identified 17 metabolites in shoots with significant interaction between treatment and genotype, of which 16 were classified as tolerant-specific and one as sensitive-specific (Fig. 5). Similarly, in roots 32 metabolites presented significant interaction of which 15 and 17 were associated with tolerant and sensitive genotypes, respectively. As was the case for the annotated metabolites, shoots and roots showed a contrasting stress response and only five unknown metabolites were significantly stress responsive in both tissues (Unknowns 37, 61, 89, 160 and 205). The identified unknown metabolites could be assigned to the same three categories as described above for the annotated metabolites and representative examples are shown in Fig. 7.

## Discussion

The aim of this study was to assess differences in the response to drought at the metabolite level in tolerant and intolerant rice genotypes. For this purpose, we have selected two traditional varieties (Dular and N22) that belong to the aus-type group and are known for their tolerance to drought, as well as P deficiency, heat and other stresses. For the intolerant genotypes, we have selected IR64 and IR74, which are well adapted to the irrigated paddy rice system and overall represent typical modern, semi-dwarf varieties.

### Changes in balances of carbon supply and plant growth under drought

The comparison of phenotypic data of plants at the vegetative stage and at plant maturity showed that all four rice varieties selected for this study responded similarly to the drought treatment by reducing shoot and root biomass and tiller numbers (Fig. 1; Additional file 2: Figure S1). Drought inhibits gas exchange and photosynthesis affecting the balance between carbon supply and plant growth as shown in Arabidopsis (Meyer et al. 2007; Sulpice et al. 2009), with starch as the major determinant of growth, in conjunction with other metabolites, such as sucrose and amino acids. In our study, drought induced the accumulation of compounds related to the central carbon metabolism (Fig. 3) suggesting that carbon utilisation for biomass formation and growth was impaired under stress. It is interesting to note that accumulation of monosaccharides in shoots was paralleled by their reduction in roots, in particular glucose, fructose and arabinose (Fig. 3). Roots are heterotrophic in nature and rely on photo-assimilates provided by photosynthesising leaves via the phloem. Reduction of these sugars and reduction in root weight, as observed in all genotypes, thus suggests that roots suffered from limited shoot C supply. This is in agreement with the finding that N22 presented the highest reduction of key metabolites (glucose, fructose, 3-P-glycerate, malate) under drought and a comparably higher reduction in root DW and lower increase in root length (Fig. 1). However, overall tolerant and intolerant genotypes all showed a similar root response to drought (Fig. 1; Additional file 2: Figure S1) showing that root plasticity and the ability to forage for deep water under drought is an important trait and highly conserved even in paddy rice.

Despite these similarities, differences in drought tolerance were obvious at plant maturity and both Dular and N22 out-yielded the irrigated varieties (Additional file 2: Figure S2). The lower grain yield in IR64 and IR74 was related to a reduced total spikelet number, rather than reduced fertility or grain size, indicative of a smaller inflorescence meristem and reduced number of floret primordia, and thus yield potential. The development of inflorescence meristems is regulated, at least in part, by cytokinin and depends on invertases and sugar supply during development (Ashikari et al. 2005; for a review see Jameson and Song 2016). However, in contrast to roots, sucrose, glucose and fructose accumulated under drought in leaves in all genotypes and sugar starvation can therefore not explain the reduced spikelet number in IR64 and IR74. It will be interesting to investigate this further and determine hormone levels and the long-term effect of drought-induced metabolites on inflorescence meristem development under drought.

### Free AA and allantoin are the main metabolites increased under drought in shoots

The metabolite analysis allowed the identification of aus-type specific metabolites that might be indicative of the pathways specifically implicated with tolerance. In fact, in shoots the most represented class of metabolites of primary metabolism were AA which accumulated to a higher level in the tolerant genotypes. Accumulation of AA is a common and well documented response to abiotic stresses (Rai 2002; Planchet and Limami 2015) although it is still a matter of debate whether this is due to increased protein degradation, decreased protein synthesis, or to enhanced AA synthesis or interconversion. However, decades of molecular studies on e.g. proline have shown that AA accumulation may have a functional role in tolerance, as first shown in drought tolerant barley by Singh et al. (1972). Since then, several studies have described proline as being an osmolyte (Yoshiba et al. 1995), a regulator of redox potential (Hare and Cress 1997), a molecular chaperone (Verbruggen and Hermans 2008; Szabados and Savoure 2010), a ROS scavenger (Mohanty and Matysik 2001) and a signalling molecule (Khedr et al. 2003). A recent field study using two contrasting rice genotypes further showed that the tolerant genotype accumulated significantly higher levels of proline under drought in roots, however, proline levels in leaves were higher in the intolerant genotype (Raorane et al. 2015).

Our data are in support of a positive role for proline under drought since it showed the second highest log2FC in shoots of all rice genotypes analysed, but a higher fold increase (5-log2FC) in the tolerant aus-type varieties compared with IR64 and IR74 (about 3-log2FC) (Fig. 4). In agreement with that, glutamate, the direct precursor of proline, was increased under drought, but this occurred in all genotypes and to about the same extent (Fig. 3). In contrast, ornithine and arginine, that can offer an alternative route for proline biosynthesis (Delauney et al. 1993; Verslues and Sharma 2010), showed a greater enrichment in the tolerant genotypes and might contribute to the observed higher log2FC-change of proline in N22 and Dular.

Aus-type genotypes also showed a greater accumulation of AA belonging to the aspartate family. Asparagine is well known to be involved in long-distance transport of nitrogen (N) and acts as a reserve of reduced N (Lea et al. 2007). Thus, asparagine may be used by plants as a reserve of N and C during stress and/or as AA storage to be used during recovery. Other components of this family associated with aus-type rice were threonine and methionine, the precursor of S-adenosyl methionine (SAM) and thus of polyamines and ethylene. Ethylene is a well-known stress hormone and any changes to this pathway that affect ethylene levels might be directly relevant for stress tolerance (for recent reviews see Müller and Munné-Bosch 2015; Salazar et al. 2015).

As the carbon backbone of methionine is derived from aspartate and the whole aspartate family of AA is tightly co-regulated (Galili et al. 2005) it is conceivable that we see parallel increases of lysine, threonine and isoleucine as well as increases of the branched chain amino acids isoleucine, valine and leucine under stress conditions. How the need for an increased flux from aspartate into the other members of the aspartate family is signalled is not clear, however, it is likely that this involves aspartate kinase which produces the common precursor aspartylphosphate (Galili et al. 2005).

In addition, the non-proteinogenic AA α-amino butyric acid (AABA), a derivate of threonine to isoleucine biosynthesis, also significantly increased in tolerant genotypes. Exogenous application of AABA to tomato plants was shown to induce the accumulation of the phytohormone ethylene (Cohen et al. 1994), suggesting that AA intermediates may indeed have important roles.

Serine and its acetylated form O-acetylserine (OAS), that are also involved in methionine biosynthesis, showed a significant differential accumulation in the contrasting rice genotypes included in this study. Interestingly, serine levels showed a higher positive log2FC-change in aus-type rice, while OAS levels were unaltered in tolerant genotypes but decreased in sensitive genotypes (Fig. 4). A recent study in *Vitis vinifera* showed no changes of OAS concentration between WW and droughted plants, while it showed altered expression of serine acetyltransferases genes (*VvSERAT1;2* up-regulated; *VvSERAT3;1* down-regulated) that convert serine to OAS (Tavares et al. 2015). OAS is then converted to cysteine, which is the precursor of glutathione (GSH), a major antioxidant, for which accumulation under drought, cold and heat shock has been well documented (Nieto-Sotelo and Ho 1986; Dhindsa 1991; Kocsy et al. 1996). Since, cysteine and GSH and methionine-downstream metabolites (SAM, spermidine, spermine, and ethylene) were not included in our study, further analyses are required to support their putative role in drought tolerance.

Interestingly, allantoin was the metabolite that presented the most significant interaction between treatment and genotypes, and also had the highest magnitude of log2FC-change in shoots (Fig. 4). Allantoin is an intermediate of purine catabolism that allows the plant to recycle N present in the purine ring. Allantoin has recently been shown to positively activate ABA and jasmonic acid in Arabidopsis, both important hormones in stress signalling (Watanabe et al. 2014; Takagi et al. 2016). It was also suggested that allantoin reduces accumulation of reactive oxygen species (ROS) under stress conditions (Brychkova et al. 2008; Watanabe et al. 2010; Irani and Todd 2016) though the exact mechanisms are unclear since allantoin did not show antioxidant activity in-vitro (Wang et al. 2012). In agreement with our data, recent findings showed that allantoin accumulates under different abiotic stresses, especially in drought tolerant genotypes in rice (Degenkolbe et al. 2013) and wheat (Bowne et al. 2011), as well as in resurrection plants (Oliver et al. 2011; Yobi et al. 2013). In contrast, allantoin was found to accumulate under drought in a sensitive barley cultivar (Chmielewska et al. 2016), suggesting that differences among plant species may exist. In the rice study by Degenkolbe et al. (2013), including twenty-one genotypes mainly originating from a Vietnamese drought breeding program, a positive correlation was revealed between allantoin levels under drought and physiological traits associated with tolerance. However, a negative correlation between levels of asparagine, serine and threonine was also reported, which is in contrast to our data but might be explained by the different drought treatments (dry-down versus 18 d drought) applied. However, the fact that allantoin was associated with tolerance independently in the present study and by Degenkolbe et al. (2013) suggests that it might indeed be a robust metabolic marker for drought tolerance justifying more in-depth studies of the pathway and its underlying genes.

### Metabolites in roots mainly show negative log2FC

Overall, the drought responsive metabolites identified in roots represent a more diverse set compared with shoots and the data were more variable, with several metabolites significant in only one of the two tolerant or intolerant genotypes and those metabolites can therefore be considered less robust (Figs. 3 and 4). That these metabolites were identified by the ANOVA is because average values for the tolerant and intolerant genotypes, respectively, were used for the analysis. However, the variability of the data also reflects the complexity of root systems and heterogeneity of soil in large pots but might also be caused by the soaking of the soil before harvest, which was inevitable for extracting intact root systems from natural soil.

Nevertheless, some metabolites showed consistent changes, especially raffinose and uridine. Raffinose has also been identified in shoots and in both tissues, intolerant genotypes showed the higher log2FC (Fig. 4). Raffinose is a soluble carbohydrate, synthesised from sucrose and galactinol (Peterbauer and Richter 2001) and the raffinose family oligosaccharides (RFOs) and biosynthetic genes are well known to differentially accumulate upon abiotic stress treatments (for a review see Sengupta et al. 2015). They are thought to play important roles in stabilizing membranes, stress signalling and as antioxidants. In Arabidopsis it has been shown that overexpression of galactinol synthase, the key enzyme for RFO synthesis, increased the concentration of galactinol and raffinose, and tolerance to ROS, salinity and chilling stress (Nishizawa et al. 2008). That, in our study, raffinose showed the higher log2FC in the intolerant genotypes (up to 4.8- versus 2.7-fold) is somewhat surprising but might in fact be indicative of the higher stress level experienced by the indica rice varieties due to the absence of protective mechanism that are present in roots of the aus-type varieties. Galactinol was identified as highly drought responsive also in this study, however, there were no significant differences between the aus-type and the irrigated varieties (Fig. 3) and galactinol is therefore not a tolerant-specific metabolite in rice.

In N22 and Dular roots, uridine was the metabolite with the highest log2FC and most significant interaction of treatment and genotype, and it was also showing a higher log2FC in tolerant shoots (Fig. 4). Uridine is a RNA-specific nucleoside containing the pyrimidine base uracil and the pentose sugar ribose. Cytidine, another pyrimidine nucleoside, as well as the products of pyrimidine catabolism (beta-alanine and its conjugate with pantoate, pantothenic acid) were also detected in this study and showed positive accumulation, although without significant genotypic differences (Fig. 3). Interestingly, the use of exogenous uridine and cytidine has been subject of a recent commercial patent, as it was shown that this enhanced plants growth under control and heat stress (45 °C) in cucumber (*Cucumis sativus*) (Cansev et al. 2014). It still remains unclear how these metabolites influence plant growth; perhaps via increasing levels of uridine-diphosphate-glucose (UDPG), which is a key metabolite involved in cell wall synthesis, glycosylation of proteins and lipids, secondary metabolism and lipid sulfonylation (for a review see Kleczkowski et al. 2010).

As seen for uridine, AABA was responsive to drought in roots and in shoots but showed a higher log2FC in the aus-types, reinforcing the notion that threonine and isoleucine might be important for tolerance (see above). The other metabolites identified in roots were less consistent and showed, with the exception of dehydroascorbic acid, negative fold changes (Fig. 4).

Interestingly, in roots, monosaccharides and metabolites representative of glycolysis and the TCA cycle, all showed negative log2FC in the aus-type varieties, notably in N22 (Fig. 4). Glycolysis and TCA cycle are involved in energy generation and are strictly connected with AA metabolism as they provide carbon skeletons required for their synthesis. Therefore, the reduction of these metabolites in roots and accumulation of AA in shoots of tolerant genotypes might suggest a controlled process to enable AA accumulation in shoots for drought protection. That this might be at the expense of root growth is indicated by the relatively lower root response in N22 compared with the other genotypes as discussed above (Fig. 1).

It is also noteworthy that the reduction of central carbon metabolites in roots was accompanied by an accumulation of the di-saccharides sucrose, galactinol, and trehalose. However, this was a general response observed in all genotypes and is therefore not tolerant-specific. Nevertheless, the importance of galactinol under drought was already mentioned above and trehalose is widely recognized for its importance in stress tolerance in different plant species (e.g. Delorge et al. 2014 and references therein). Importantly, the signalling molecule trehalose-6-phosphate (T-6-P) has recently been shown to significantly increase yield and recovery from drought in Arabidopsis, maize and wheat and is now being tested as an agro-chemical (Nuccio et al. 2015; Griffiths et al. 2016).

### Many unknown metabolites are highly drought responsive

About two-third of the metabolites that we identified as responsive to drought and associated with tolerance were unknowns (Fig. 5). Identifying a large number of unknowns is not surprising and it is estimated that plants have up to 1 million metabolites (Saito and Matsuda 2010) while commercial libraries generally include only a few thousand. It will therefore require a major effort and investment to reveal the nature of these molecules and the underlying pathways.

For now, our data may therefore serve as another example for the untapped potential of molecules that accumulate under drought and for the genetic diversity within rice, and other crops. Virtually all unknown metabolites that were significant in shoots based on the two-way ANOVA showed a higher magnitude of change in the aus-types (Fig. 5), as was also observed for the known metabolites, especially allantoin and AA as discussed above (Fig. 4). Similarly, a greater number of both, known and unknowns, showed a negative log2FC in roots, with the known metabolites mainly associated with glycolysis and TCA. However, in contrast to the known metabolites, many unknowns showed a higher-fold positive change in the intolerant genotypes, including MST 197, which is the most significant metabolite identified in the two-way ANOVA across the entire experiment (Fig. 5). Interestingly, the main differences between tolerant and intolerant genotypes are under WW conditions where the aus-types had about 100 times the levels of MST 197 compared to the intolerant lines, while under drought the difference was only 10 times (Fig. 7).

### Metabolites show different drought response patterns

Our results showed that for the majority of significant metabolites the determinant of the difference between tolerant and sensitive genotypes was the magnitude of log2FC, rather than the abundance (Fig. 6). For some metabolites, such as allantoin and the unknowns MST 89 and 197, the higher log2FC and association with tolerant genotypes is indeed determined at WW control conditions, rather than under stress (Figs. 6 and 7). This was also indicated by the PCA analysis (Fig. 2) which showed some separation of the tolerant and intolerant genotypes under WW conditions, especially in shoots. To determine whether or not metabolites have a role in conferring tolerance it therefore seems important to assess the magnitude of change and the absolute concentration. It has for example been shown that, in a drought tolerant rice variety (TKM-1), proline levels under drought increased from 250 to 1350 μg g^−1^ DW (5.4-fold), while in a sensitive variety (Sabarmati) it increased from 755 to 900 μg g DW^−1^ (1.2-fold). Despite the fact that proline levels under WW conditions were about 3-times higher in the sensitive variety, the authors proposed a correlation of proline accumulation and drought tolerance based on the differences in fold-change (Mali and Mehta 1977).

Metabolites correlating with beneficial traits have the potential to be used in breeding (see Matros et al. 2017 and reference therein). However, in contrast to DNA-based markers, metabolites are much less robust since they are responsive to the environment, and might be tissue-specific and developmentally regulated. Rather than using metabolite markers, it will therefore be important to develop DNA-based markers targeting the genes underlying the differential response of a given metabolite. This is, however, only possible if the genes and regulators of the pathway are well known and allelic variation between tolerant and intolerant genotypes exist. However, metabolites can also be used for statistical association with genomic regions, i.e. mQTL mapping, which opens opportunities to employ unknown metabolites for breeding as well as known metabolites (Fernie and Schauer 2009; Matsuda et al. 2012). As more and higher quality *de-novo* assembled genome sequences become available (Huang et al. 2012; Schatz et al. 2014; Du et al. 2017), mQTL mapping will also facilitate the identification of the underlying genes and pathways, which might be genotype specific as is being shown for an increasing number of agronomically important genes (Xu et al. 2006; Hattori et al. 2009; Gamuyao et al. 2012).

Metabolites that show differences under WW control conditions, such as allantoin or MST 197, might be generally more eligible for high-throughput screens in breeding programs because they do not require stress treatment but could be predictive of the stress tolerance capacity. Curiously, the example from proline and our data on allantoin suggest that in some cases, plants with a low concentration of a given metabolite should be selected as a prerequisite for the required high magnitude of change desirable under stress.

## Conclusions

The comparison of traditional aus-type rice with irrigated varieties allowed us to identify tolerant-specific metabolites that accumulate in shoots and/or roots under drought. These metabolites have protective roles as osmolytes (proline), N storage molecule (asparagine), stress signalling (allantoin) and growth enhancer (uridine). Therefore, together with the underlying genes and pathways, they are interesting targets for in-depth studies on their role in drought tolerance. Our data suggest that the protective function of certain metabolites under stress may depend on the magnitude of the accumulation upon stress rather than on abundance (e.g. allantoin and unknown MST 197). In addition, for certain tolerance-related metabolites we show that the difference among genotypes is already pre-determined under control conditions. If a causal relationship to stress tolerance can be demonstrated, these metabolites may be suitable candidates for high throughput screens in breeding programs, avoiding the need to screen under stress. Mapping of both, known and unknown metabolites in conjunction with the availability of *de-novo* genome sequences of tolerant genotypes will enable us to gain access to the underlying genes and pathways and devise strategies for crop improvement.

## Additional files

Additional file 1: Table S1. Experimental information and data on metabolite peaks. (XLSX 1006 kb)

Additional file 2: Supplemental Figure S1 to Figure S4. (PPTX 3810 kb)

Additional file 3: Table S2. Log2 ratios of Drought vs Well-Watered conditions per genotype and 2-way ANOVA. (XLSX 138 kb)
