# Supplementary material for: Exploring traditional aus-type rice for metabolites conferring drought tolerance
Source: Rice (N Y). 2018 Jan 25;11:9. doi: 10.1186/s12284-017-0189-7 (PMC5785456; doi:10.1186/s12284-017-0189-7)
Supplement: Supplementary file 2 — Supplemental Figure S1 to Figure S4. (PPTX 3810 kb) [file 12284_2017_189_MOESM2_ESM.pptx]

## Slide 1
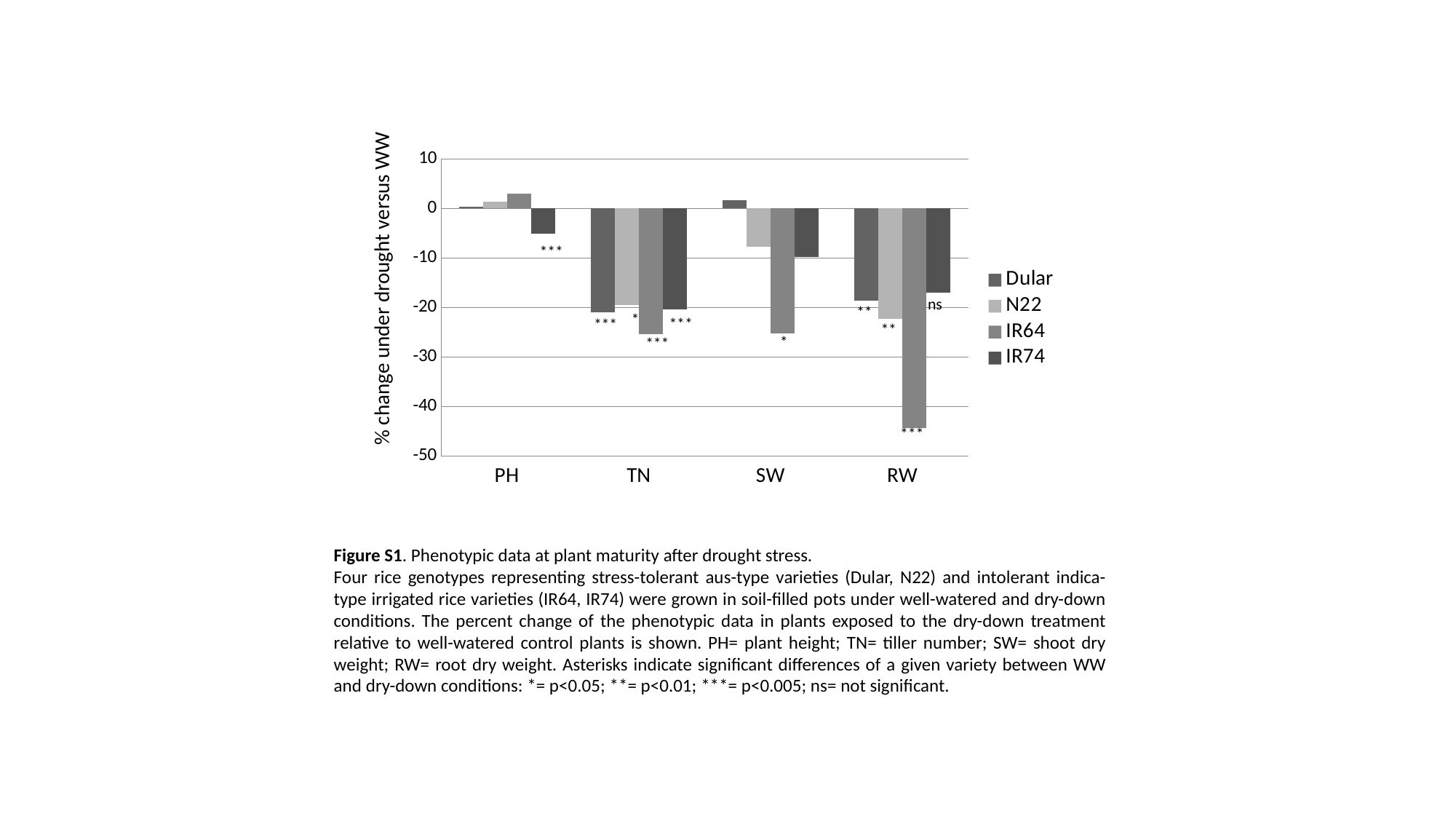

### Chart
| Category | Dular | N22 | IR64 | IR74 |
|---|---|---|---|---|
| PH | 0.397971127584867 | 1.329051824101342 | 3.071284125379163 | -5.119980993109986 |
| TN | -20.8955223880597 | -19.51219512195121 | -25.40983606557377 | -20.35398230088495 |
| SW | 1.711335136464214 | -7.701157690371758 | -25.19326485227153 | -9.742957746478863 |
| RW | -18.6731799558777 | -22.29809277136802 | -44.42613948380011 | -16.94464944649445 |***
ns
**
*
***
***
**
*
***
***
% change under drought versus WW
Figure S1. Phenotypic data at plant maturity after drought stress.
Four rice genotypes representing stress-tolerant aus-type varieties (Dular, N22) and intolerant indica-type irrigated rice varieties (IR64, IR74) were grown in soil-filled pots under well-watered and dry-down conditions. The percent change of the phenotypic data in plants exposed to the dry-down treatment relative to well-watered control plants is shown. PH= plant height; TN= tiller number; SW= shoot dry weight; RW= root dry weight. Asterisks indicate significant differences of a given variety between WW and dry-down conditions: *= p<0.05; **= p<0.01; ***= p<0.005; ns= not significant.

## Slide 2
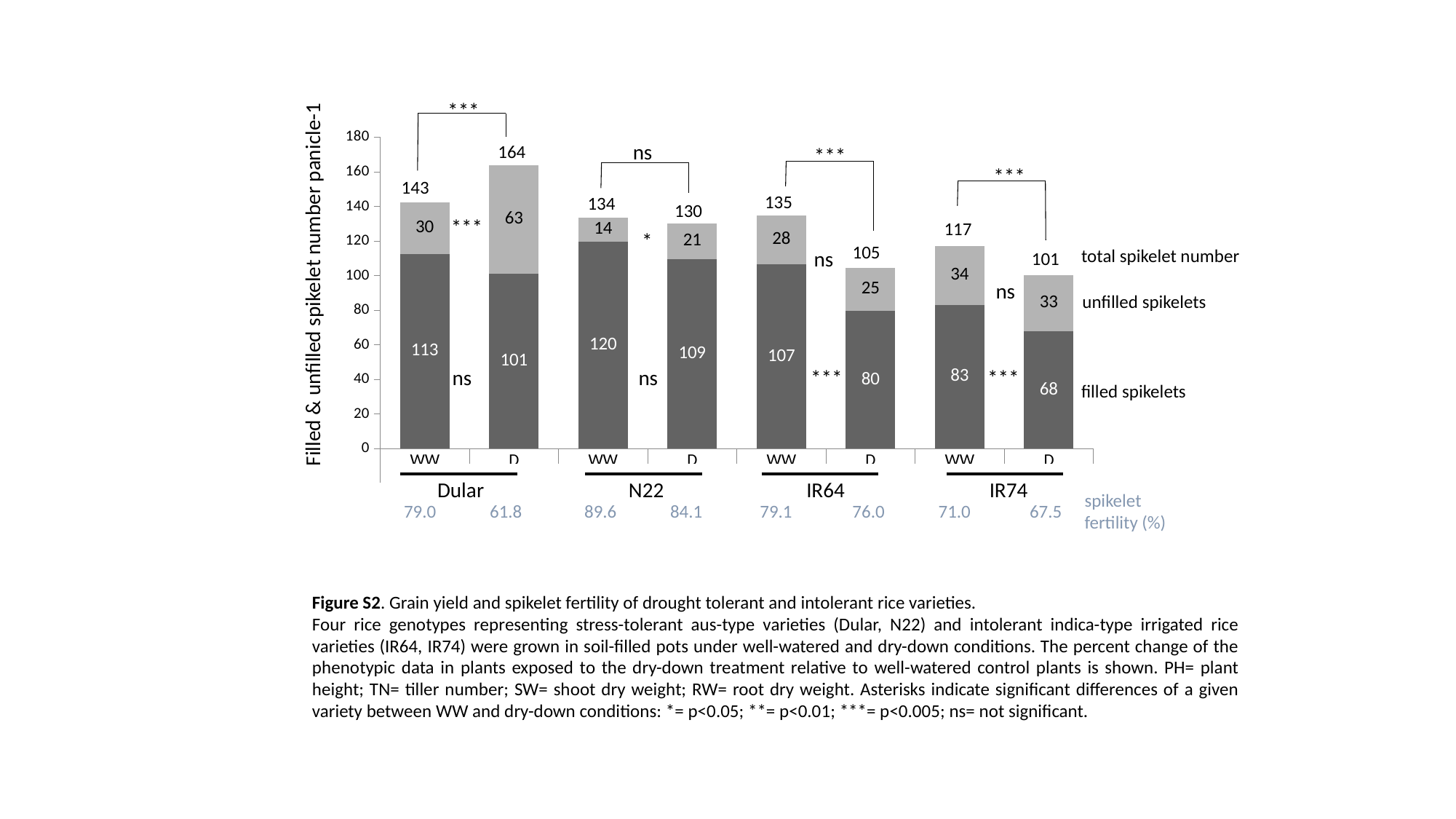

***
### Chart
| Category | av filled grain 5 pan | av unfilled grain 5 pan |
|---|---|---|
| WW | 112.65 | 29.9 |
| D | 101.275 | 62.575 |
| WW | 119.75 | 13.875 |
| D | 109.425 | 20.75 |
| WW | 106.55 | 28.225 |
| D | 79.525 | 25.1 |
| WW | 83.25 | 33.95 |
| D | 67.725 | 32.625 |ns
164
143
135
134
130
117
105
101
***
***
***
*
total spikelet number
ns
Filled & unfilled spikelet number panicle-1
ns
unfilled spikelets
ns
ns
***
***
filled spikelets
Dular
N22
IR64
IR74
spikelet
fertility (%)
79.0
61.8
89.6
84.1
79.1
76.0
71.0
67.5
Figure S2. Grain yield and spikelet fertility of drought tolerant and intolerant rice varieties.
Four rice genotypes representing stress-tolerant aus-type varieties (Dular, N22) and intolerant indica-type irrigated rice varieties (IR64, IR74) were grown in soil-filled pots under well-watered and dry-down conditions. The percent change of the phenotypic data in plants exposed to the dry-down treatment relative to well-watered control plants is shown. PH= plant height; TN= tiller number; SW= shoot dry weight; RW= root dry weight. Asterisks indicate significant differences of a given variety between WW and dry-down conditions: *= p<0.05; **= p<0.01; ***= p<0.005; ns= not significant.

## Slide 3
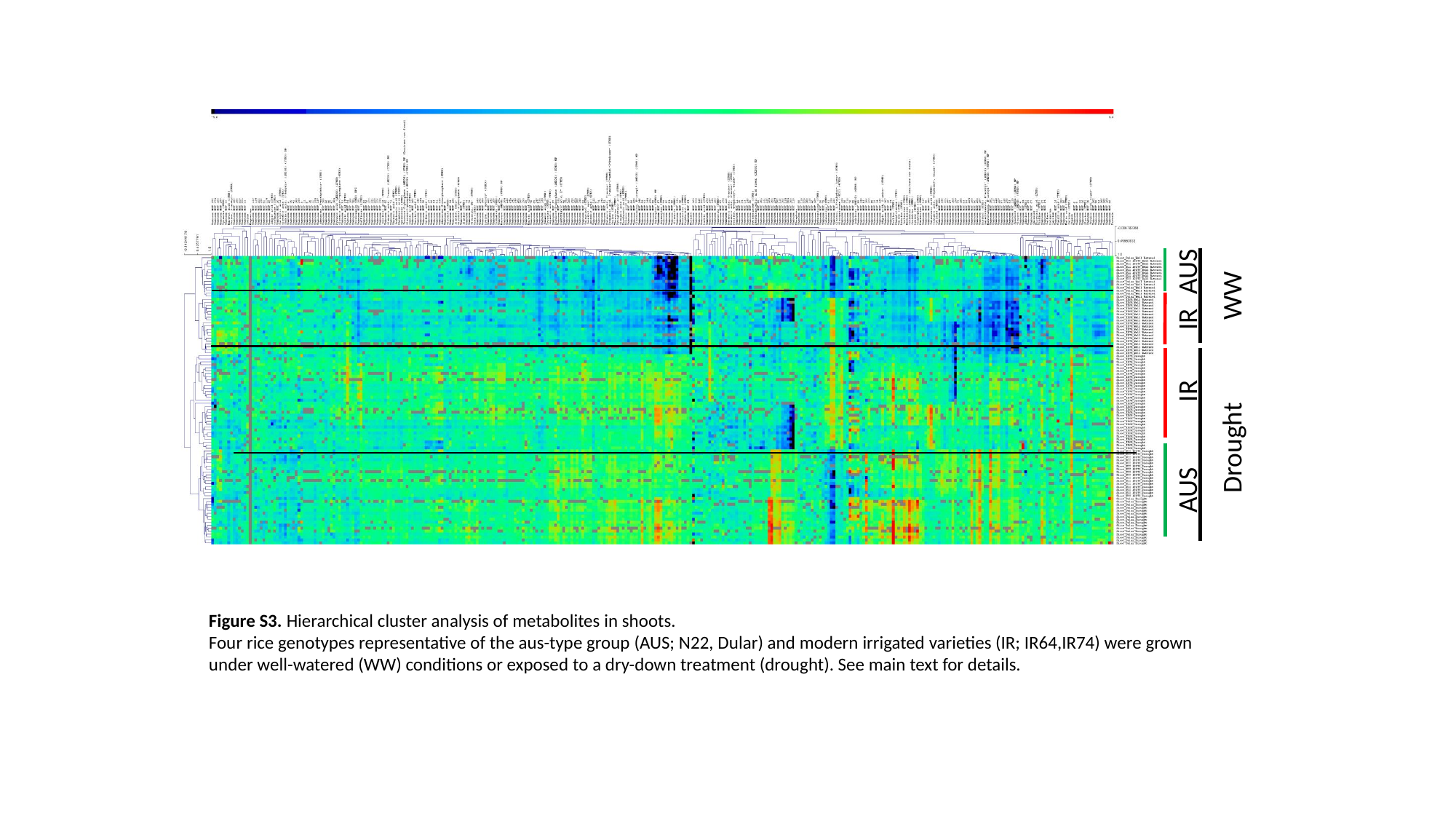

AUS
WW
IR
IR
Drought
AUS
Figure S3. Hierarchical cluster analysis of metabolites in shoots.
Four rice genotypes representative of the aus-type group (AUS; N22, Dular) and modern irrigated varieties (IR; IR64,IR74) were grown under well-watered (WW) conditions or exposed to a dry-down treatment (drought). See main text for details.

## Slide 4
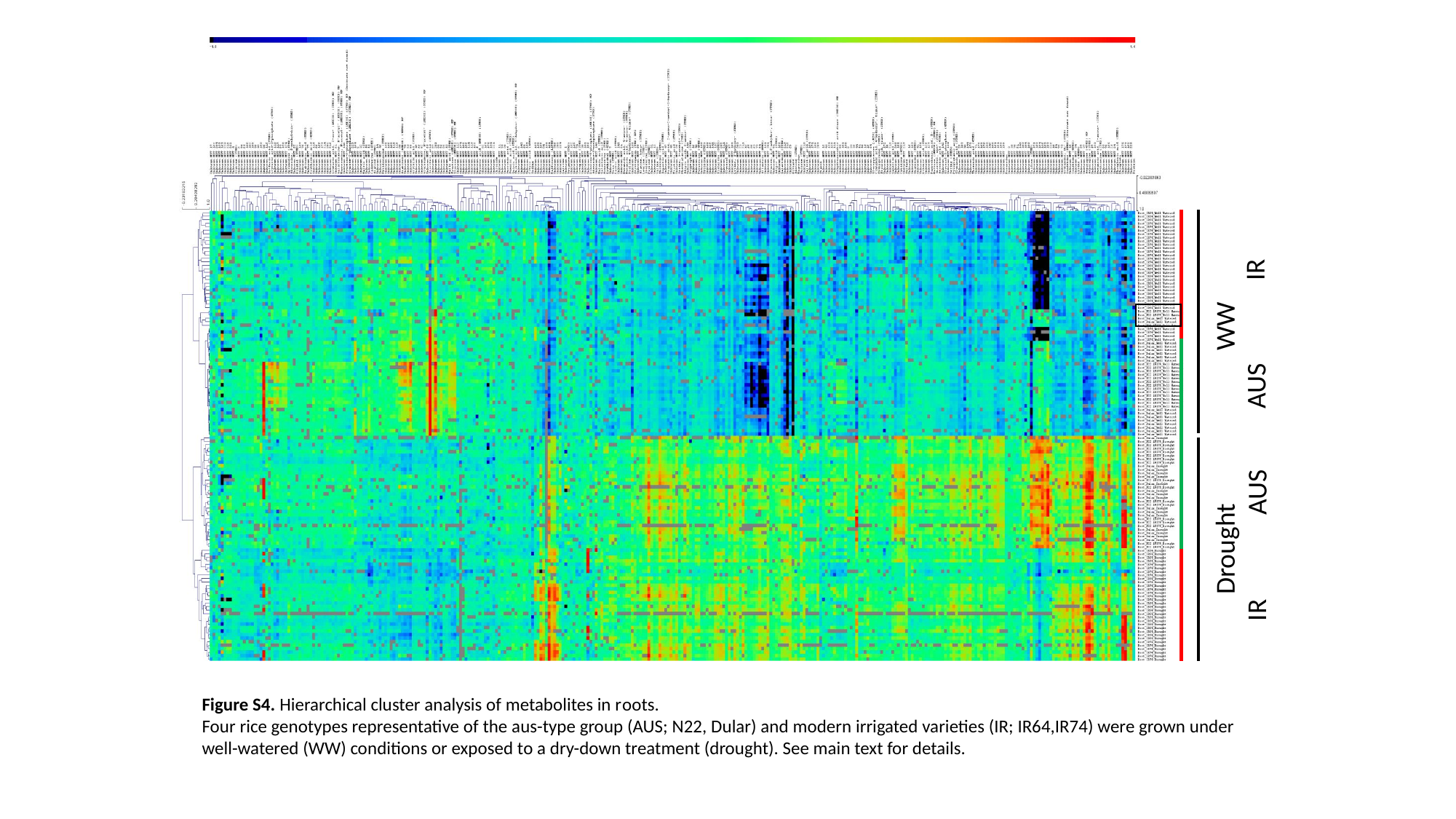

IR
WW
AUS
AUS
Drought
IR
Figure S4. Hierarchical cluster analysis of metabolites in roots.
Four rice genotypes representative of the aus-type group (AUS; N22, Dular) and modern irrigated varieties (IR; IR64,IR74) were grown under well-watered (WW) conditions or exposed to a dry-down treatment (drought). See main text for details.
